# Effect of Fire Frequency on the Flammability of Two Mediterranean Pines: Link with Needle Terpene Content

**DOI:** 10.3390/plants10102164

**Published:** 2021-10-12

**Authors:** Bastien Romero, Anne Ganteaume

**Affiliations:** Institut National de Recherche pour L’agriculture, L’alimentation et L’environnement (INRAE), 13182 Aix-en-Provence, France; bastien.romero@inrae.fr

**Keywords:** forest fire, climate change, terpenes, fuel flammability, *Pinus* sp.

## Abstract

Flammability is a major factor involved in Mediterranean plant evolution that has led to the diversity of fire-related traits according to fire regimes and fire-adaptive strategies. With on-going climate change, new fire regimes are threatening plant species if they do not adapt or acclimate. Studying flammability and terpene content variation according to the different fire frequencies in the recent fire history represents a great challenge to anticipating the flammability of ecosystems in the near future. The flammability of shoots and litter as well as the needle terpene contents of two pine species with different fire adaptive strategies (*Pinus halepensis* and *Pinus sylvestris*) were measured according to two fire modalities (0 vs. 1–2 fire events over the last 60 years). Results showed that, regardless of the species and the fuel type, flammability was higher in populations having undergone at least one past fire event even when factors influencing flammability (e.g., structural traits and hydric content) were considered. The terpene content did not vary in *P. sylvestris’* needles according to the fire modality, but that of sesqui- and diterpenes was higher in *P. halepensis’* needles sampled in the “Fire” modality. In addition, associations made between flammability and terpene content using random forest analyses indicated that the terpene molecules differed between fire modalities for both species and fuel types. The same results were obtained with significant terpenes driving flammability as were highlighted in the PLS analyses, especially for *P. halepensis* for which enhanced shoot flammability in the “Fire” modality agreed with the adaptive strategy of this species to fire.

## 1. Introduction

The flammability of a plant species can be considered a niche construction trait [1,2,3]. Indeed, in fire-prone ecosystems, fire acts as a natural disturbance, controlling age, structure, and species composition [4,5,6,7,8,9]. For several decades, the increase in fire risk in the Mediterranean basin has been directly linked to an increase in temperatures [10,11,12]. With current climate change, the temperatures will continue to increase along with a decrease in precipitations that will lead to an increase in the frequency of extreme fire events in some regions such as southeastern France [13,14]. The flammability of a plant species could be impacted by these changes, which could affect entire forest ecosystems [15,16].

Fire is a complex process involving many factors, but fuel flammability is commonly assessed by the following four components [17,18]: (i) ignitability, (ii) combustibility, (iii) sustainability, and (iv) consumability. These flammability components are used in most studies assessing plant flammability [19,20] even though these studies have been criticized recently due to the high correlation between the flammability variables belonging to different components [21] or to their scarce empirical support [22]. Even if the flammability components are well defined, in some cases, plant flammability can differ among scales (e.g., from fuel particle to litter) [23] and among fire-resilient and fire-resistant species according to their flammability strategies [24]. For example, some pine species considered as less flammable, due to their low flammability at the plant level, have highly flammable litter in order to increase the flame spread and, therefore, to decrease the fire time of residence [21]. Many plant traits are known to have a high impact on plant flammability such as the fuel moisture content (FMC), the leaves/needles structural characteristics (e.g., thickness, surface-to-volume ratio, etc.), and the fuel structure (e.g., the shoot or fuel bed bulk density) among others [23,25,26,27]. These traits differ among species that present different fire-adaptive strategies, but they can also differ within the same species according to fire frequency [28,29]. In [28], the authors found that the flammability of *Ulex parviflorus* increased with fire frequency due to the change in the plant structure (e.g., bulk density). These results are consistent with the hypothesis stated in [30] that suggests the possible heritability of flammability.

Burning experiments in the laboratory are useful to assess small scale flammability. Furthermore, controlling factors that affect flammability (e.g., FMC) highlight the effects of other factors that would be masked otherwise [21]. Mediterranean regions are characterized by the presence of plant species differing in fire-related traits according to the fire regime (defined by the frequency and intensity of fires) [31,32,33,34,35,36]. In ecosystems undergoing low intensity fires (i.e., surface fires), most fire-adapted species are qualified as resistant (e.g., *Pinus sylvestris*) [37] or as “fast-flammable” species [24]. These species present traits such as thick bark and/or self-pruning [38,39] that reduce the impact of fire on these trees. However, some fast-flammable species can have high litter flammability due to their low litter compactness that increases the flame rate of spread and, therefore, decreases the flame residence time [24]. Most species growing in areas undergoing intense fire (i.e., crown fires), referred to as fire-embracer species, have an adaptive strategy based on high flammability, or “hot-flammable” [24]. The fire-embracer species, such as *Pinus halepensis* or several *Fabaceae* species, have functional traits that enhance post-fire regeneration, such as releasing their canopy seed bank after a fire or having a soil seed bank.

Plant chemical content has often drawn attention in fire literature [40,41,42]. Among the most studied molecules impacting flammability are the volatile or semi-volatile terpenes because of their chemical characteristics (e.g., low flash point) and because they are produced by many species growing in different climates [43,44]. Terpenes are involved in many ecological processes such as the response of a plant to biotic (e.g., pest attack) or abiotic (e.g., drought, light, or temperature) stresses [45,46,47]. These molecules can be stored inside plant tissues through specialized structures such as resin ducts or trichomes [48,49] or even directly emitted once synthetized. Three major families are related to the number of carbon atoms in the molecule composition: (i) monoterpenes (C10), (ii) sesquiterpenes (C15), and (iii) diterpenes (C20). Several studies dealt with the relationship between terpene content and flammability [40], sometimes with contrasted results. Some of them [50,51] found no clear evidence on the impact of monoterpenes on flammability while others [20,48] found that mono- and sesquiterpenes enhanced the flammability of leaf litter of several Mediterranean species (e.g., *P. halepensis*, *Rosmarinus officinalis*). More recently, [52] and [53] also showed that terpene concentration had a contrasted impact on flammability according to the terpene family and molecules involved, the flammability variables measured, and the species studied. These results are consistent with those of [40], which showed that different terpene molecules could have different impacts on flammability. However, all these studies considered terpene molecules in their analyses only if they were highly concentrated (i.e., a concentration higher than a specific level or that explained a significant part of the total amount of terpenes), and/or if these studies correlated flammability variables to only a single terpene compound. Furthermore, only a few flammability variables were considered, and the burning protocols used did not, for instance, consider fuel structure. Since species flammability can be considered adaptive [24], it could be of interest to study how it can be affected by a variation in fire frequency. In addition, knowing that terpenes contained in plant tissues can affect flammability while still having a genetic basis [54,55], studying the link between terpenes and flammability according to fire frequency for two species with different adaptive strategies represents a great challenge.

Addressing the gaps previously highlighted, two common Mediterranean pine species presenting different adaptive strategies to fire (fire-resistant and fire-embracer) were studied in the present work. The goals were, therefore, to compare (i) the intraspecific variation of shoot and litter flammability while also considering structural key traits well known as flammability drivers, and (ii) the terpene content (focusing on mono-, sesqui-, and diterpenes) according to two fire modalities (“Fire” vs. “No-Fire”). The last goal of this work was to link flammability to groups of terpene compounds in order to explain the differences observed in flammability between fire modalities. The fire frequency was expected to have an impact on the species’ flammability according to their fire adaptive strategy, with higher flammability for the shoot and litter of the fire-embracer species as compared to the fire-resistant species. In addition, the effect of the terpenes on each flammability variable was supposed to differ among the species, the fire modalities, and the flammability components with the possibility of having the opposite effect.

## 2. Results

### 2.1. Variation of Flammability According to the Fire Modality

#### 2.1.1. Shoot Flammability

**Figure 1:**
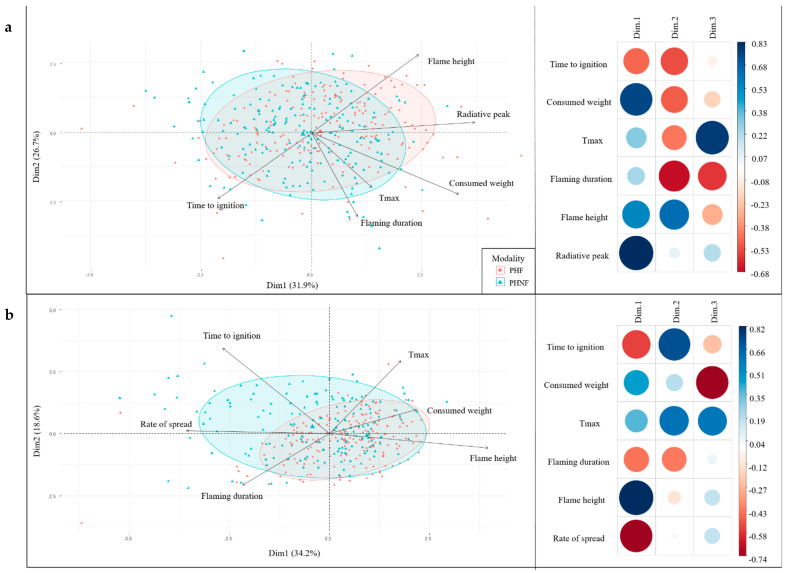
Biplots of principal component analyses (only the two first components (Dim) are shown) illustrating relationships between flammability variables (Tmax: maximum temperature) and fire modalities for *P. halepensis*’ shoot (**a**) and litter (**b**) flammability. Populations sampled in the high Fire recurrence modality (PHF) are circled in blue, and populations sampled in the No-Fire modality (PHNF) are circled in orange. The correlation plots indicate the regression coefficient of each trait on each component: the darker and larger the circle color and size, the stronger the correlation.

**Figure 2:**
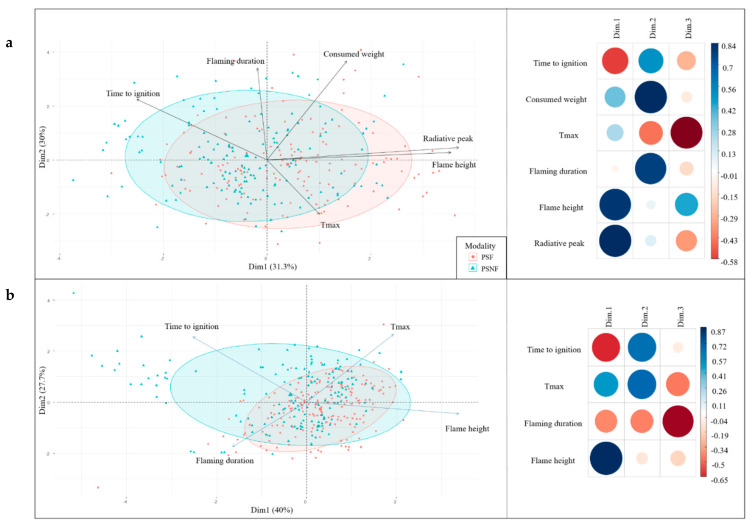
Biplots of principal component analyses (only the two first components (Dime) are shown) illustrating relationships between flammability variables (Tmax: maximum temperature) and fire recurrence modalities for *P. sylvestris*’ shoot (**a**) and litter (**b**) flammability. Populations sampled in the high Fire recurrence modality (PSF) are circled in blue, and populations sampled in the No-Fire modality (PSNF) are circled in orange. The correlation plots indicate the regression coefficient of each trait on each component: the darker and larger the circle color and size, the stronger the correlation.

The principal component analyses (PCA) performed on the flammability variables measured for *P. halepensis* and *P. sylvestris*’ shoots showed that the three first components explained more than 75% of the total variance (77.43% and 79.99%, respectively, Figure 1a and Figure 2a). For both species, the radiative peak variable was mostly related to the first component, the flaming duration variable to the second component, and the maximum temperature variable was best related to the third component while the time-to-ignition variable was split between the two first axes. For *P. halepensis*, the flame height was split between the two first components while for *P. sylvestris*, it was best characterized by the first component. The consumed weight of *P. halepensis*’ shoots was mostly represented on the first component but was displayed on the second component for *P. sylvestris*’ shoots. Moreover, the PCA results showed that there was no clear segregation between the “Fire” and the “No-Fire” populations, which were mostly distributed along the first axis (although the data was more scattered for *P. sylvestris*).

The simple linear regression analyses performed on both modalities and relating each flammability variable to significant cofactors (to highlight the possible impact of the FMC, the surface-to-volume ratio (SVR), and the bulk density on flammability; see Table S1) indicated that, for both species, each flammability variable was significantly related to at least one cofactor. For *P. halepensis*, the SVR was significant for the entire set of flammability variables, except for the radiative peak and the flame height. *P. sylvestris*’ flammability was mostly related to the FMC, which was significant for all of the flammability variables, except for the flaming duration. A one-way analyses of variance (ANOVA) performed on the cofactor-corrected flammability variables showed that only a few tests were significantly different between the “Fire” and “No-Fire” populations, regardless of the species (Table 1 and Table 2). Regarding *P. halepensis*, the time-to-ignition (F = 17.42; *p* < 0.0001) and the flame height (F = 10.89; *p* = 0.0011) variables had higher values for the “No-Fire” populations, whereas flaming duration was longer for the “Fire” populations (F = 8.90; *p* = 0.0030) (Table S2). Regarding *P. sylvestris*, the maximum temperature and the radiative peak variables were higher for the “Fire” populations as compared to the “No-Fire” populations (F = 25.28; *p* < 0.0001 and F = 11.92; *p* = 0.0006, respectively) (Table S2).

#### 2.1.2. Litter Flammability

The PCAs performed on both species’ datasets indicated that the three first components explained more than 67% of the total variance (69.79% for *P. halepensis* and 40.01% for *P. sylvestris*, Figure 1b and Figure 2b) with eigen values higher than one. For both species, the flame height was positively related to the first component as was the rate of spread for *P. halepensis.* For this species, the time-to-ignition variable characterized the second component and the consumed weight variable characterized the third one. The flaming duration variable was split between the two first axes and the maximum temperature variable between components two and three. For *P. sylvestris*, the maximum temperature mostly characterized the second component, and the flaming duration was best related to the third component. The time-to-ignition variable was split between axes one and two. As for the shoot flammability, these PCAs showed that there was no clear segregation between the fire modalities, regardless of the species (for both species, the data related to the “No-Fire” modality were the most scattered along axis one).

The litter flammability variables were only related to the SVR (the flaming duration and flame height variables for *P. halepensis* and the time-to-ignition, flaming duration, and flame height variables for *P. sylvestris*, Table S3) because the bulk density and the FMC were controlled for these burning experiments. Regarding *P. halepensis*, the time-to-ignition (F = 34.48; *p* < 0.0001), rate of spread (F = 14.75; *p* = 0.0001), and flaming duration (F = 5.23; *p* = 0.0232) variables presented lower values for the “Fire” populations in contrast to the consumed weight (F = 10.17; *p* = 0.0016) and flame height (KW = 21.93; *p* < 0.0001; Table 3 and Table S4) variables. The time-to-ignition (KW = 10.63; *p* = 0.001) and the maximum temperature (F = 23.09; *p*< 0.0001) presented higher values for the *P. sylvestris*’ “Fire” populations, whereas the flaming duration (KW = 18.94; *p* < 0.0001) and flame height (F = 107.38; *p* < 0.0001) values were higher for the “No-Fire” populations (Table 4 and Table S4).

### 2.2. Variation of Terpene Content According to the Fire Modality

**Figure 3:**
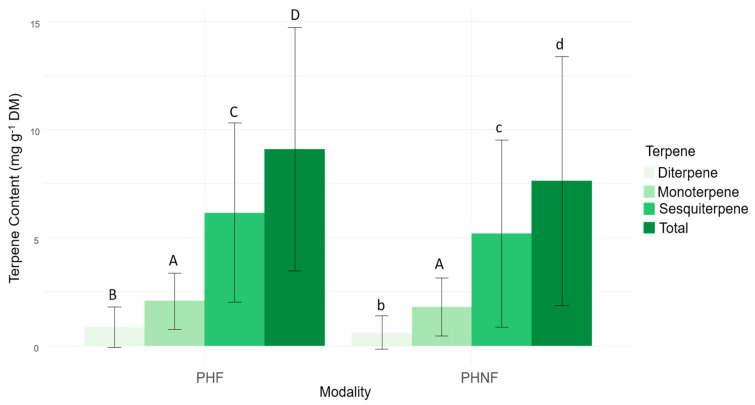
Variation of monoterpene, sesquiterpene, diterpene, and total terpene concentrations (means ± SD, in mg g^−1^ DM) according to the fire modality for *P. halepensis*. PHF: *P. halepensis’* “Fire” modality and PHNF: *P. halepensis’* “No-Fire” modality. Letters indicate differences between terpene families according to the fire modality (A > a, B > b, C > c, and D > d), Kruskal-Wallis test, *p* < 0.005.

For *P. halepensis*, the terpene content analysis showed that 23 terpene compounds were detected in the majority of the samples, divided into monoterpenes (11), sesquiterpenes (10), and diterpenes (2) (see Table S5). The sesquiterpene content was significantly higher than that of the monoterpene and the diterpene contents (5.68 mg g^−1^ DM vs. 1.93 mg g^−1^ DM and 0.86 mg g^−1^ DM, respectively, Kruskal-Wallis, KW = 463.6, *p*< 0.0001). The five most abundant molecules were β-caryophyllene, elemol, α-pinene, caryophyllene-oxide, and α-muurolene, which represented 63.6% of the total amount of the terpene content for *P. halepensis*. It is worth noting that β-caryophyllene (2.40 ± 1.42 mg g^−1^ DM), by itself, represented 28.5% of the total amount of terpenes. The sesquiterpene, diterpene, and total terpene contents were significantly higher for the “Fire” populations (Figure 3).

For *P. sylvestris*, the terpene content analysis showed that 44 terpene compounds were detected in the majority of the samples (see Table S6), divided into monoterpenes (19) and sesquiterpenes (25). The monoterpene content was higher than that of the sesquiterpene content (5.02 mg g^−1^ DM vs. 3.66 mg g^−1^ DM, one-way ANOVA, F = 44.35, *p*< 0.0001). The five most abundant molecules were α-pinene, β-cadinene, camphene, β-myrcene, and “unknown sesquiterpene 35,28”, which represented 48.6% of the total amount of the terpene content for *P. sylvestris*. No significant differences in the terpene content was found between the “Fire” and the “No-Fire” populations, regardless of the terpene family.

### 2.3. Terpenes Linked to Flammability According to the Fire Modality

Regardless of the species, groups of molecules identified by random forest analyses differed between the fire modalities. For the *P. halepensis’* shoot flammability (the flammability variables were cofactor-corrected when necessary), the analyses indicated that 21 different terpene molecules were linked to flammability in total (Table 1 and Table S7). Considering each modality separately, 19 different terpene molecules were related to the flammability of the “Fire” populations and 13 to the flammability of the “No-Fire” populations. Regardless of the fire modality, flammability was mainly related to the sesquiterpenes (10 compounds) in contrast to the monoterpenes (9 compounds) and the diterpenes (2 compounds). Β-caryophyllene was related to each flammability variable for the “Fire” populations, except for the time-to-ignition and the flame height variables, as was α-muurolene for the “No-Fire” populations, except for the time-to-ignition variable. Thunbergol was the only molecule related to flammability (the consumed weight variable) for the “No-Fire” populations, but it was not related to that of the “Fire” populations.

*P. halepensis*’ litter flammability was mostly equally related to the monoterpenes and the sesquiterpenes (Table 3 and Table S8). Only one diterpene (cembrene) explained the flaming duration and rate of spread variables for the “Fire” populations as well as the time-to-ignition, flaming duration, and consumed weight variables for the “No-Fire” populations. The terpene molecules related to flammability were more diverse for the “Fire” populations with 17 different molecules as compared to the “No-Fire” populations with 16 different molecules, accounting for 20 compounds in total. This result showed that more molecules were linked to the “Fire” populations’ flammability than to that of the “No-Fire” populations’, but the number of terpene molecules linked to each flammability variable in the “No-Fire” populations was higher for the flame height (8 vs. 7), rate of spread (7 vs. 1), and consumed weight (8 vs. 4) variables. Ocimene and β-cadinene explained most of the flammability variables for the litter of the “Fire” and “No-Fire” populations, respectively.

Random forest analyses performed on *P. sylvestris*’ shoot flammability variable according to the fire modality indicated that 23 terpene molecules were linked to flammability in total. Twenty different molecules (Table 2 and Table S9) were related to flammability for the “Fire” populations as well as 9 molecules for the “No-Fire” populations. Even though the diversity in the sesquiterpenes was higher than that of the monoterpenes, the majority of the terpene molecules associated with flammability were the monoterpenes. It is worth noting that the “unknown sesquiterpene 35,28”, one of the most concentrated molecules, was not related to flammability (non-significant results) in contrast to the other less concentrated molecules, such as borneol for the maximum temperature in the “Fire” and “No-Fire” populations or for the flaming duration in the “Fire” populations. Regarding the intersection between the Boruta and VSURF packages, only two terpene molecules were common to the “Fire” and “No-Fire” modalities (borneol and β-myrcene for the maximum temperature variable).

Regarding *P. sylvestris*’ litter, 29 terpene molecules were linked to flammability, regardless of the variable and the fire modality (Table 4 and Table S10). Regarding the “Fire” populations, 23 different molecules were related to flammability (β-bourbonene being the compound in common with all of the variables) while there were only 11 molecules for the “No-Fire” populations. For the “No-Fire” modality, only the flaming duration variable was not related to any terpene molecules. Contrary to the shoot flammability variable, *P. sylvestris*’ litter flammability was mainly related to the sesquiterpenes, especially for the “Fire” populations. Several highly concentrated molecules (e.g., β-cadinene, α-pinene, unknown sesquiterpene 35,28, and camphene) were not linked to the litter flammability in contrast to the results on the shoot flammability. Several molecules were common to the “Fire” and “No-Fire” modalities, such as α-terpineol and linalool. However, when comparing the terpene molecules linked to the litter flammability of the “Fire” and “No-Fire” populations, it is worth noting that the 18 molecules involved in the flammability of the “Fire” populations were not related to those of the “No-Fire” populations.

### 2.4. Influence of Terpene Molecules on Flammability According to Fire Modality

Significant correlations were highlighted in the partial least squares (PLS) regression analyses linking, for both of the species and fuel types, each flammability variable to the terpene molecules among those extracted from the random forest analyses (i.e., the significant molecules). The groups of significant terpene compounds mostly differed among their flammability variables as well as between the fire modalities for a given variable. Regardless of the species and the fuel type, the total number of significant compounds was mainly higher in the “Fire” modality than in “No-Fire”. Sometimes, compounds belonging to the same terpene family could have an opposite effect on a given variable, making it more difficult to highlight the prevailing effect on the flammability variables (i.e., less contrasted results). It is worth noting that, in some cases, the same molecule could have an opposite effect on the same flammability variable between the two fire modalities (e.g., elemol on the maximum temperature variable for *P. halepensis*’ litter). For both *P. halepensis* and *P. sylvestris*, regardless of the fuel type, the regression coefficients of the significant molecules presented higher values for the “Fire” modality (but, overall, lower for the latter species). The same result was observed for the strengths of the relationships (R^2^) (Table 1, Table 2, Table 3 and Table 4; Tables S11–S14).

For *P. halepensis*’ shoots, regardless of the fire modality, different compounds belonging to the mono- and sesquiterpenes (as well as two diterpene compounds for the “No-Fire” modality only) were significantly related to flammability, the former mostly mitigating flammability in contrast to the latter. (Table 1 and Table S11). For *P. halepensis*’ “Fire” litter, the effect of the sesquiterpene and mostly monoterpene compounds on flammability was less contrasted (often with a negative effect) than for the shoots. The pattern was clearer for the “No-Fire” modality with the monoterpene compounds, mostly enhancing their flammability in contrast to the sesqui- and diterpenes (Table 3 and Table S12).

Regarding *P. sylvestris*’ “Fire” populations, the shoot flammability was mostly driven by the monoterpene compounds (mostly enhancing flammability) while for the “No-Fire” modality, the effect of the terpene compounds was less contrasted (Table 2 and Table S13). For *P. sylvestris*’ “Fire” litter, the sesqui- and monoterpene compounds mainly drove the flammability (the former with a negative effect) while, for the “No-Fire” modality, the results were less contrasted with the flammability driven by the monoterpenes and sesquiterpenes. (Table 4 and Table S14).

## 3. Discussion

### 3.1. Effect of the Fire Frequency on Flammability

The descriptive analyses results showed that no clear pattern of flammability emerged between the populations sampled in the different fire modalities, regardless of the fuel type and the species. The lack of clear differentiation could be caused by too little contrast between the two fire modalities (0 vs. one-to-two fires), given that only the recent fire history was considered (no data was available before 1959, at the earliest, for a major part of the study area). As already mentioned previously, flammability is a complex phenomenon that must be broken down to be better assessed [21,22]. According to the components, results showed several significant differences in flammability between the two fire modalities for both species studied. Regardless of the species, the shoots from the “Fire” populations were mostly more flammable (higher ignitability and sustainability for *P. halepensis* and higher combustibility for *P. sylvestris*) than those of the “No-Fire” populations. *P. halepensis*’ litter flammability was mostly higher for the “Fire” populations in contrast to that of *P. sylvestris* (lower flame height, shorter flame residence, and longer time-to-ignition). This agreed with the fire-adaptive strategy of these two pine species. Indeed, higher flammability for *P.halepensis*’ “Fire” populations is consistent with the ecological niche creation hypothesis advanced by several authors [1,2,24]. The litter of *P. halepensis*’ populations sampled in this fire modality easily ignited and produced taller flames as compared to the litter sampled in the “No-Fire” modality. This latter characteristic is important to consider regarding the probability of flame propagation from the understory vegetation to the canopy [21]. The production of serotinous cones by *P. halepensis* is an important functional trait, but cones need a higher temperature emitted by the fire to induce the melting of the waxes and resins that seal the cones to succeed in opening them [56,57]. In contrast, *P. sylvestris* is a resistant species and its strategy is to survive fire [37]. However, the shoot flammability of the “Fire” populations was higher than that of the “No-Fire”, which could be in contradiction with this work’s first hypothesis that argued that the shoot flammability of a resistant species will decrease in trees that grow after a fire event. However, this contradiction was displayed by only two variables (the results for the others were non-significant). Regarding the litter, a shorter flaming duration as well as a lower flame height in the “Fire” populations is an advantage for *P. sylvestris* because these characteristics, respectively, reduce the impact of high temperatures (even if the maximum temperature is higher) because of the decreased flame residence time as well as the lower probability of fire propagation to the canopy.

Fuel flammability is driven by numerous factors such as fuel moisture content and leaf physical properties. The effect of these factors on flammability have been studied for a long time [50,58,59,60], but these plant characteristics can vary strongly according to environmental conditions and individual ontogeny including water availability, temperature, and tree age [29,61]. Furthermore, some of these traits are related to fire and could vary according to the fire frequency. For example, [28] found that *Ulex parviflorus’* bulk density increased with fire frequency, enhancing the flammability of this fire-embracer species.

### 3.2. Variation in Terpene Content and Composition According to the Fire Frequency and Their Links to Flammability

Another goal of this study was to show if there were, for both species, any differences in their terpene-molecule content according to the fire modality, in their composition related to the fuels’ flammability, and among these molecules, which best drove flammability in each case.

Regarding the variation of the terpene content according to the fire modality, results showed that the two species did not present the same pattern. Indeed, the terpene content did not vary between the fire modalities for *P. sylvestris* in contrast to *P. halepensis*, for which the sesquiterpene and diterpene content as well as the total content was higher for the “Fire” populations. The higher content of molecules possibly increasing flammability [20] in the *P. halepensis*’ “Fire” populations is consistent with this species’ fire adaptive strategy that requires fire to open the serotinous cones since terpenes are also involved in the resin production needed for the production of these cones [44,62]. This result also highlighted that the role of these molecules in this strategy could pass from one generation to the next. Moreover, [3] underlined that the impact of fire as a selective pressure could have played a significant role in the abundance of plants emitting and storing terpenes. Indeed, the terpene content is considered to have a genetic base [20,54,55] and, therefore, is heritable through generations as these secondary metabolites were considered among the most evolvable traits [63]. The chemical analyses did not highlight any diterpene compounds in *P. sylvestris* and only two compounds were identified in *P. halepensis*, which is rather low compared to previous studies, such as [62]. This could be due to a different method being used for the GC-MS analyses (the sample analysis was 20 min shorter in the current work than in [64], which impacted the detection of the heaviest molecules).

Regarding the determination of which groups of terpene compounds were associated with flammability, the random forest analyses showed that molecules belonging to the three terpene families were related to flammability, but these were not necessarily the most concentrated regardless of the fire modality, the species, or the fuel type. This result confirms that highly concentrated molecules do not always drive flammability, and that all of the terpene families can have an impact on flammability [53]. Most importantly, for a given variable, the groups of molecules linked to a species’ fuel flammability differed according to the fire modality (e.g., the flaming duration variable for *P. halepensis*’ shoots or for the time-to-ignition variable for *P. sylvestris*’ shoots), meaning that there could be clusters of significant terpene compounds specific to a given fire modality. In addition, terpene-single-compound diversity that was significantly linked to flammability was always higher for the “Fire” populations, regardless of the species and the fuel type.

Regardless of the fire modality, most flammability variables were driven by one or several molecules (regardless of the terpene family), which agreed with previous studies [52,64]. These compounds mostly differed between the modalities and among the variables. Furthermore, the same molecule could have an opposite effect on the same flammability variable according to the fire modality (e.g., elemol on the maximum temperature variable for *P. halepensis*’ litter). Low-concentration molecules could also have a strong impact on flammability, such as camphene (0.008 ± 0.004 mg g^−1^ DM), which was the main shoot flammability driver for *P. halepensis*’ “No-Fire” populations, decreasing flame height. It is worth noting that the relationship between terpenes and flammability was weaker for the “No-Fire” modality, regardless of the species and the fuel type, suggesting that other factors could have a stronger effect on flammability. For *P. halepensis*, the effects of significant compounds driving shoot flammability (either the monoterpenes or the sesquiterpenes) could differ between the fire modalities with flammability being enhanced in the “Fire” modality in contrast to the “No-fire” modality, which agrees with this species’ fire adaptive strategy, while the results were less contrasted for litter. It is worth noting that the diterpene compounds were only among the significant drivers in the “No-Fire” modality, regardless of the fuel type. For *P. sylvestris*, the effect of the terpene compounds on flammability was not clear, regardless of the fuel type, for the “No-Fire” modality. Indeed, a lower number of the variables presented significant relationships with terpenes for this modality, which could mean that, in this case, terpenes are not the best drivers of flammability. Regardless of the fuel type, monoterpene compounds enhanced flammability for the “Fire” modality. Often, the opposite effect between compounds of the same family on a given variable could be highlighted, regardless of the species and the fuel type, making the results less contrasted. This result, consistent with previous studies [40,53,64], confirms the complexity of the relationships between flammability and chemical compounds, and, especially, terpenes.

## 4. Materials and Methods

### 4.1. Area and Species Studied

**Figure 4:**
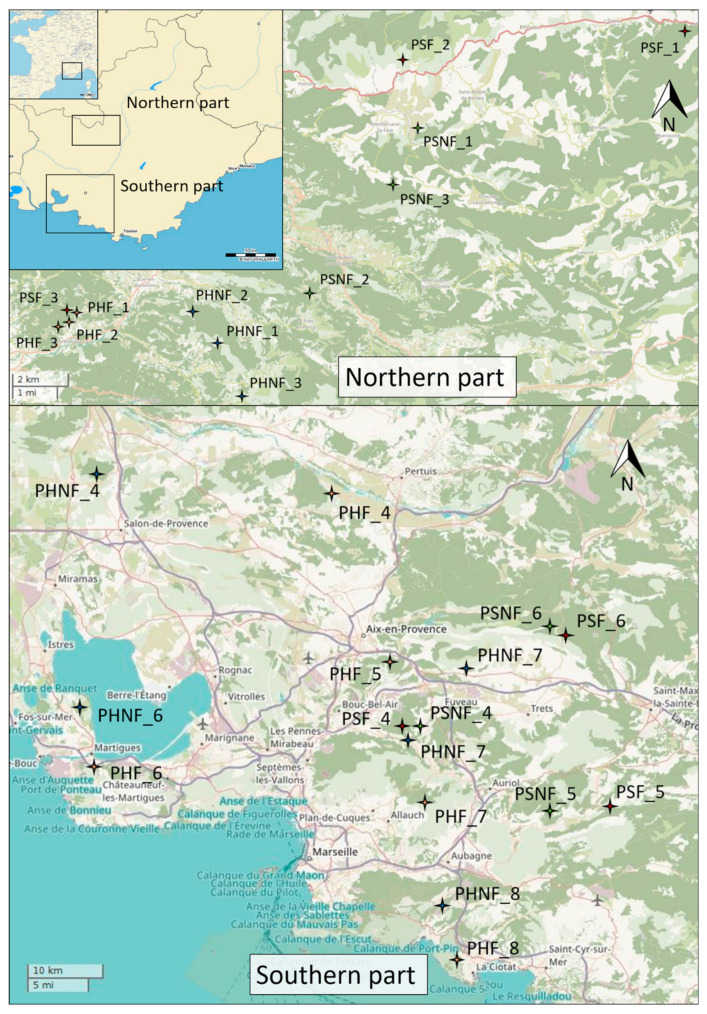
Map of the study area with a focus on the northern and southern sampling plots (PH, *P. halepensis*; PS, *P. sylvestris*; F, “Fire” populations; NF, “No-Fire” populations).

Southeastern France is the region the most affected by fires (with 4657 fires and more than 21,000 ha burned during the 2010–2020 period according to the regional fire database Prométhée; available online: www.promethee.com (accessed on 8 October 2021)). Generally, this region is considered to have a typical Mediterranean climate with mild and humid winters, hot and dry summers, and calcareous soils (i.e., limestone). However, Mediterranean areas also have a high diversity of sub-climate, vegetation, and landscape characteristics, inducing different fire regimes on a finer scale [9]. In order to account for this variability, sampling sites were located on a North-South gradient in the Provence region (in southeastern France, Figure 4), which corresponded to a gradient from the meso- to sub-Mediterranean climates [65] and, therefore, to a gradient in fire regimes. The northern part of the study area is characterized by a sub-Mediterranean climate [65] with one or two dry months. The fire activity in this area is quite low (e.g., there were 576 fire events from 2010 to 2019 in the administrative district of Drôme, which is located in the northern part of the study area), involving mainly low-intensity surface fires. The southern part of the study area is characterized by a meso-Mediterranean climate with three or four dry months [65]. Fires in this landscape are more frequent (i.e., there were more than 4100 fires events from 2010 to 2019 in the administrative district of Bouches-du-Rhône) and are more intense (involving mainly crown fires).

The most common species in the entire study area are *Quercus ilex* L. and *Quercus pubescens* Willd. as well as *P. halepensis* and *P. sylvestris*, with the latter found at higher elevation [66]. In this study, the two pine species *P. halepensis*, and *P. sylvestris* were chosen because they are obligate seeders that are not able to resprout after a fire; the post-fire recruitment is therefore only based on seeds [43]. This type of recruitment facilitates faster generation turnover and makes the species more sensitive to changes in fire frequency [67]. Furthermore, these two species have different adaptive strategies to fire (fire-embracer vs. fire-resistant, respectively). *P. halepensis* can be considered as a hot-flammable species, according to the fire strategy developed by [24] and is adapted to infrequent stand-replacing fires. Its post-fire regeneration strategy is based on the creation of an aerial canopy seed bank, referred to as “serotiny,” where seeds are protected in serotinous cones and then released after fire events. The heat shock induced by the high flame temperatures induces the cone to open [68,69]. In this species, serotiny and early flowering (when they are around 10 years old according to [70,71,72]) reflect the fire-embracer strategy as these characteristics allow for successful post-fire recruitment in the event of frequent fires [68]. With climate change, *P. halepensis*’ biogeographical area is expected to expand toward the northern area of the study, which is the current boundary of its distribution area [73]. *Pinus sylvestris* is considered as a fast-flammable or a fire-resistant species, as defined in [24,37], and is adapted to recurrent low- to medium-intensity fires that correspond to surface fires [74,75]. This species has developed traits that allow for resistance to such fires (e.g., height, self-pruning, and thick bark) [37]. The geographical distribution of this species is related to lower temperatures and/or higher elevations as compared to that of *P. halepensis*, and the populations are very well established in the northern part of the study area as well as at higher altitudes and on north-facing slopes in the South. In contrast to the previous species, *P. sylvestris*’ biogeographical area is expected to recede according to climate change, ensuring stand mortality that will increase the fire risk in these areas.

### 4.2. Sampling Plan

Even if the Mediterranean basin is prone to recurrent fire events, knowing the exact fire history of a location over the last century is sometimes difficult, especially when many species do not present with fire scars. In Southeastern France, the exhaustive monitoring of fire events was established approximately 60 years ago, providing reliable information on fire frequency, but not on fire intensity, for this period. In this study, the recent fire history (between 1959 or 1973, depending on the area, and 2018) was reconstructed using different regional fire databases (the fire database of Office National des Forêts/Direction Départementale des Territoires et de la Mer, has been georeferencing fire perimeters since 1961, and the Prométhée fire database has been recording fires since 1973, at the earlier) as well as satellite image analyses to refine fire perimeters (using the open-source QGIS software; https://www.qgis.org/, accessed on 8 October 2021). These databases were used for sampling tree populations according to the fire modality. For both species, half of the populations were sampled in unburned areas (“No-Fire” modality, i.e., no fire in the last 47–59 years, depending on the database) and the other half in areas that had been subjected to at least one fire (“Fire” modality; i.e., one fire or more in the last 47–59 years, depending on the database). All of *P. halepensis*’ “Fire” populations were sampled in areas having experienced stand-replacing fires, thereby having completely burned during these past events. In contrast, in *P. sylvestris*’ “Fire” populations, some trees resisted the past fires, inducing a post-fire recruitment based solely on tree survival (only trees younger than the age of the last fire were sampled in order to account for trees that grew after the fire event). When populations had undergone more than one fire event (i.e., up to 2 fires in *P. halepensis*’ populations), those with at least a ten-year interval since the last fire were selected to be sure that the trees that regenerated after the most recent fire were sexually mature [72]. Samples were taken from pairs of “Fire” and “No-Fire” populations that were located between two and eight kilometers apart in order to avoid geographical segregation and genetic divergence caused by drift. All trees of the same species were sampled in the same age range (22.5 ± 2.2 years for *P. halepensis* and 20.3 ± 6.0 years for *P. sylvestris*) and were mature (i.e., cones were observed in the canopy), dominant, healthy trees. Forests were mapped according to the presence (i.e., recent forest) or absence (i.e., ancient forest) of past land use (former agricultural lands), which has been found to affect soil nutrients [76]. Populations were sampled in homogeneous past land use in order to mitigate the impact of soil conditions on trait variations. In total, 16 *P. halepensis* populations of 20–30 trees and 12 *P. sylvestris* populations of 20–30 trees were sampled according to the fire modality.

### 4.3. Flammability Measurements

Burning experiments were performed on shoots and litter of both species. Regarding shoots, the same protocol was used for both species, whereas two different protocols were designed for *P. halepensis* and *P. sylvestris* due to the low litter accumulation under the trees of the latter species. Regardless of the species or fuel type, at least one flammability variable per flammability component was measured.

#### 4.3.1. Shoot Flammability

Shoots were sampled during July 2019 for *P. halepensis* and September 2019 for *P. sylvestris*, between 12 pm and 2 pm to avoid hydric and chemical content variation throughout the day. The shoots sampled had to be healthy, well developed, and south exposed as well as located in the middle of the canopy. The authors in [28] underlined the importance of the FMC in the intraspecific variation of flammability. To eliminate possible differences in shoot moisture among indiviuals/populations sampled in different sites, the shoot-conditioning protocol prior to burning described in [28] was followed. In this protocol, shoots, once sampled, were stored in plastic bags for 24 h at 5 °C and then for 12 h in the dark at an ambient temperature before burning. However, this parameter can still have a significant impact on shoot flammability, so the moisture content of the leaves of each individual shoot was measured just before burning (see below) so it could be considered in the statistical analyses. For each tree, four 20 cm shoots (half without needles) were calibrated for burning. Three samples were used for the flammability assessment while the fourth was used to obtain the weight of the needles needed for the calculation of the shoot bulk density (which was used as a cofactor in the analyses; see below), along with the shoot volume. The FMC was measured on a 5 g subsample of needles dried at 60 °C for 48 h prior to burning in order to obtain their dry weight, which is necessary to calculate the moisture content according to the following Equation (1):

FMC = (Mf(g) − Md(g))/(Md(g))(1)

where Mf(g) represents the fresh fuel mass, and Md(g) represents the dry fuel mass. The moisture content of needles, and not that of the entire shoot, was measured because only this part was burned during the experiments.

**Figure 5:**
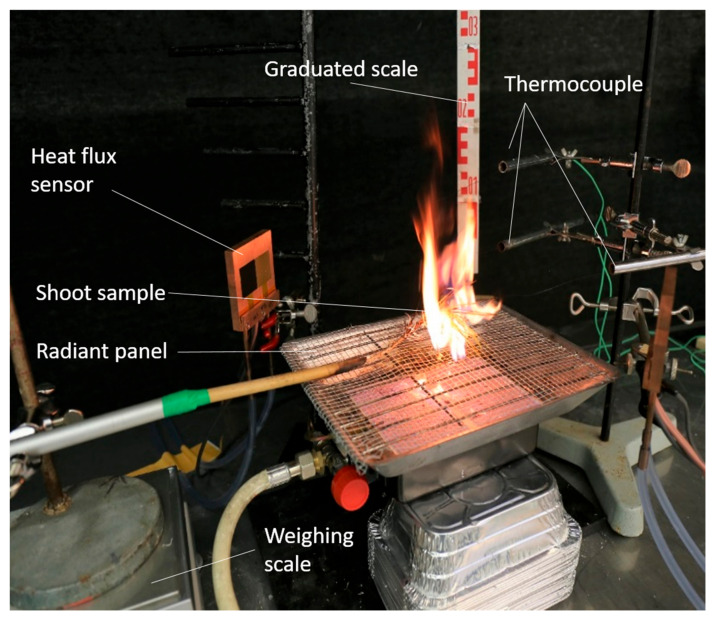
Picture of the burning device used for shoot sample in both species.

Samples (N = 1140 for *P. halepensis* and N = 900 for *P. sylvestris*) were burned using a 4 kW radiant panel (switched on 30 min before burning tests in order to have a steady heat release) as an ignition source (Figure 5) due to its stability in terms of heat release compared to other types of ignition sources [77]. The radiant panel was placed horizontally, allowing the heat to radiate upward, while the shoot sample was placed 1 cm above the center of the radiant-panel grid. Three 0.25 mm type K (Chromel-Alumel) thermocouples, two located 5 cm above the radiant-panel grid and one 10 cm above the center, were linked to a data logger (ALEMO 2590 Ahlborn, Ahlborn Mess- und Regelungstechnik GmbH, Holzkirchen, Germany) to record the temperature variation (one record per second) during the burnings to assess the combustibility component. The temperature used in the analyses was the mean temperature between all of the thermocouples. In addition to the temperature, the combustibility was also assessed using two heat flux sensors (Captec Entreprise, Lille, France), which measured the convective and total fluxes that were placed at 20 cm from the center of the radiant panel. The mean value of both heat flux sensors was used to calculate radiative flux (W m^−2^) released per second (i.e., total flux–convective flux) and to extract the maximum radiative flux reached (i.e., radiative peak). The flame height (FH in cm, combustibility component) was measured using a ruler placed behind the radiant panel and a camera (CANON 77D, 50 mm optic, Canon, Tokyo, Japan) placed in front of the burning device (i.e., located 50 cm from the shoot with an angle of 30°) to film the burning tests for the flame height assessment (combustibility component). For assessing the consumability component, the fuel weight before and after burning was measured to calculate the weight (g) consumed during the burning.

Using a chronometer, the time-to-ignition of the shoot was measured once the sample was positioned above the radiant panel (i.e., time-to-ignition (TTi), ignitability component). The time until the end of the burning, or flaming duration (FD) (i.e., when the flame went out, sustainability component), was calculated by subtracting the total time of the burning from the time-to-ignition.

#### 4.3.2. Litter Flammability

For both species, litter sampled in situ was sorted, removing leaves belonging to other species as well as other plant particles in order to keep only the healthy needles of the studied species. This sorting allowed the assessment of the flammability of a given species’ needles only. However, the litter amount was higher under *P. halepensis* than under *P. sylvestris*, so the burning protocol had to be adapted according to the species. The FMC of litter samples was not estimated because, as cited above, litter samples were oven-dried for 48 h at 60 °C before the burning experiments (i.e., no moisture remained).

##### *P. halepensis* 

**Figure 6:**
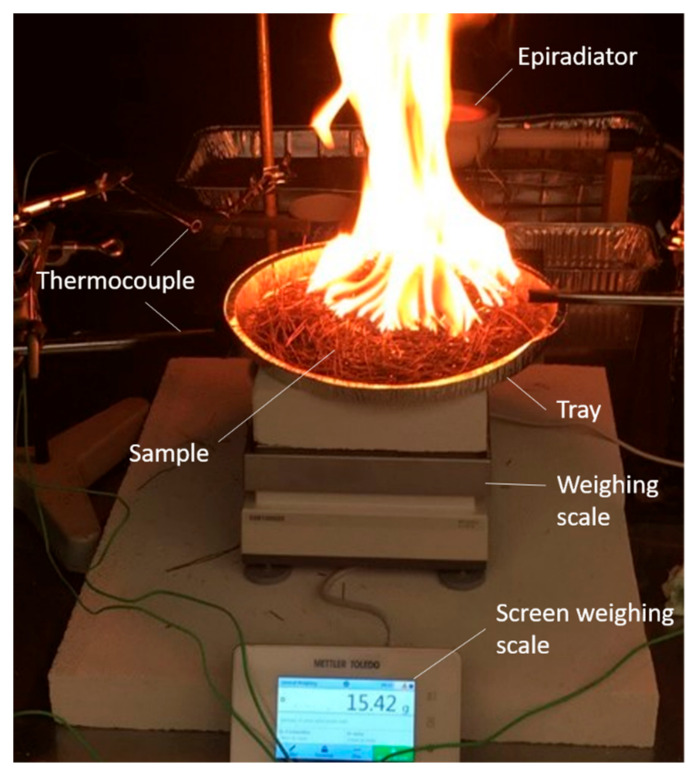
Picture of the burning device for *P. halepensis’* litter.

For each tree, three burnings of 20 g were performed (in total, 1140 burnings) in a 27 cm diameter circular aluminum tray (Figure 6). In addition, the sample thickness and weight had to be homogeneous among burnings (as it corresponded to the height of the aluminum tray) because the bulk density is an important factor inducing flammability variation [78].

Trays were disposed on an electronic scale in order to measure the sample weight loss during the burning and to calculate the consumed fuel weight. The temperature emitted by the flames was measured with four type K 0.25 mm (Chromel-Alumel) thermocouples linked to a data logger and placed according to the four cardinal points, five centimeters above the litter sample and at equal distance between the center and the edge of the tray (i.e., 13.5 cm from the center). The data logger measured the temperature every second, and the data were averaged during post-treatment. The ignition source was a standard (i.e., calibrated) flaming pine wood piece (2 cm × 2 cm × 1 cm) called a “firebrand” that was placed in the center of the sample [78]. This method ensured an identical ignition for all of the samples. The “firebrand” was ignited using a 500 W epiradiator and, after ten seconds of the flaming phase, it was dropped off in the center of the litter sample. Once touching the litter, the chronometer was switched on to record the time-to-ignition (TTi) and the flaming duration (FD). After the litter ignition, the pine cube was removed from the sample.

##### *P. sylvestris* 

**Figure 7:**
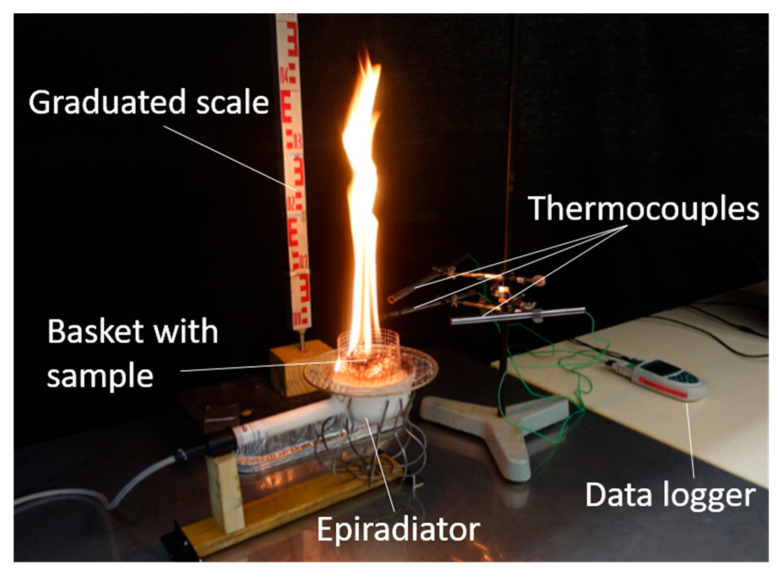
Picture of the burning device for *P. sylvestris* litter.

A circular (i.e., 80 mm diameter and 55 mm height) aerated basket made with a 5 mm mesh metal grid was designed for the litter burning experiments of this species (Figure 7). This method provides adequate oxygen circulation through the litter. As for the litter of *P. halepensis*, three burnings per tree were performed, representing, in total, 900 burnings (450 for each fire modality). A constant weight of 2 g litter (previously oven-dried at 60 °C for 48 h), respecting a 5mm-fuel bed thickness, was harmonized for all the samples. To allow ignition, the basket was placed on the grid that was positioned on a 500 W radiant disk (10 cm diameter epiradiator), respecting a 1 cm distance between the sample and the surface of the epiradiator, in order to account for convection phenomena. Here again, three type K 0.25 mm (Chromel-Alumel) thermocouples located 5 mm above the basket (i.e., 70 mm above the epiradiator surface) were used to record temperature every second. The same protocol as the one described for the shoot flammability assessment was used in order to measure the time-to-ignition (TTi), the flaming duration (FD) and the flame height (FH). Eventually, 100% of the litter was consumed during each burning, so the consumed weight was not considered in the analyses.

### 4.4. Flammability Cofactors

In order to better link terpenes and flammability according to the fire modality, common driving factors used in flammability studies (see data analysis) were considered for the statistical analyses. Even if shoot conditioning was performed to reduce the FMC range (90.5 ± 8.9% for *P. halepensis* and 103.2 ± 10.5% for *P. sylvestris*) among the populations and the trees, this factor could still have an impact on flammability and had to be considered in the analyses to avoid any possible overriding effect on terpene content. In addition to the FMC, two other traits (litter needle surface-to-volume ratio and shoot bulk density) possibly driving flammability were also measured for the statistical analyses. Along with the packing ratio, the bulk density is an important factor to be considered in a flammability assessment [22,78]. Regarding the litter samples for both species, the fuel weight and thickness were the same for all the samples to induce the same litter bulk density. Regarding the shoot samples, width and length were measured in order to calculate the volume of the cylinder representing the shoot (taking into account only the part with needles). The bulk density (kg m^−3^) was then calculated by dividing the weight of the needles (previously weighed as explained above) by the shoot volume.

The leaf surface-to-volume ratio (SVR, m^−1^) is also commonly used as a potential driver of flammability [58,79,80]. The SVR was calculated for five needles per tree based on the measurements of the needle thickness, length, and elongation [81] using the WinSEEDLE program (WinSEEDLE 2014, © Regent Instruments Inc., Canada, accessed on 8 October 2021) and according to the following formula (2):

SVR = (4/T) ((1 − e/2) (4/π)H + 2B/πW)(2)

where T is the thickness (m), W is the width (m), e is the elongation (e = 1 T/W), and H is a function of the elongation (H = e/(2 − e)^2^).

### 4.5. Terpene Content Analysis

For each tree of both species, approximatively 3 g of healthy needles were collected randomly on several shoots and then stored in individual paper bags at −80 °C in order to stop the terpene metabolism.

As in [53] for terpene content extraction, 500 mg of needles per tree were ground up using liquid nitrogen and put into 4 mL of extraction solution (i.e., cyclohexane and dodecane). After a 5 min ultrasound agitation, the vial content was filtered (PTFE—0.22 µm filter) and transferred into a 3 mL vial for analysis. Dodecane (alkane) was not naturally present in the samples, and so it was used as an internal standard (i.e., knowing its quantity and retention time). Gas chromatography (GC-MS System 7890B, Agilent Technologies, Montpellier, France) was used to determine the terpene concentration and the composition of the samples. One mL of solution was injected at 250 °C into a capillary column (HP-5MS, Agilent J&W GC Columns, Agilent Technologies, Montpellier, France) at a constant flow (i.e., 1 mL min^−1^, helium as the carrier gas). At the beginning of the process, the temperature was 40 °C and increased at a rate of 3 °C min^−1^ up to 300 °C during the analysis. A solvent delay of 5 min was observed, and the total run time was 70 min.

The terpene molecule identification was carried out using MassHunter spectrometry software. The identification was based on the molecule retention time (RT) and on their mass spectrum. All of these identifications were checked by comparing their retention index (RI) to common libraries [82]. The retention index was calculated using the alkanes injected at each session (3):

RI(mol) = 100 × X(a) + (RT(mol) − RT(α))/(RT(β) − RT(α)) × 100(3)

where α is alkane before the molecule, β is alkane after the molecule, X is carbon number, and RT is retention time.

Several dilutions of many authentic reference compounds were performed then analyzed in the GC-MS in order to impute the response factor of each terpene family in the spectrometry software. Because this work was carried out on fresh samples, the sample dry mass had to be calculated first before the terpene concentration per dry mass unit.

Eventually, we kept only the molecules present in at least 80% of the samples and for which the identification was certain. In addition, the choice was made to remove the molecules β-myrcene and guaiol from *P. halepensis*’ analyses because of the concentration aberrations (concentrations higher than 20 mg g^−1^ DM in several samples) that were certainly due to the fact that the calibration curves of the authentic reference compounds were not adapted for these two compounds.

### 4.6. Data Analysis

The statistical analyses were performed on each species and fuel type (shoot or litter), first using principal component analyses (PCA) as the preliminary analyses in order to select the most important flammability variables (explaining at least 70% of the variation) and check the flammability pattern according to the fire modality.

For both species and fuel types, simple linear regressions were performed in order to highlight correlations between the cofactors (FMC, SVR, and/or bulk density) and the flammability variables. When the cofactors explained a significant proportion of the variability of a given flammability variable, the residuals from the multiple regression, performed with all the cofactors considered, were used as cofactor-corrected data of this variable. This helped us to highlight, when possible, the effect of the fire modality without the bias of the above-mentioned factors when their effect on flammability was significant [20,29,53]. Each flammability variable (corrected or not) was tested according to the fire modality using a one-way ANOVA, followed by a Student’s *t* test or a Kruskal-Wallis test, followed by a Bonferroni test when the normality of data was not possible.

The differences among the concentrations of the terpene families according to the fire modality were tested using the Kruskal-Wallis test, followed by the Bonferroni test because of the non-normality of the data distribution. In order to select a molecule or a group of molecules linked to flammability, random forest (RF) analyses were performed [83,84]. Commonly used in genetic studies, RF analyses are very useful in selecting explanatory variables linked to the variation of a response variable and, in this study, were able to account for several important factors [85]. For both species, RF analyses were performed considering the molecules identified in the samples only if they were present in at least 50% of the trees sampled and at a minimum content threshold of 0.0001 mg g^−1^ DM. The RF analyses were carried out with two different R packages, Boruta [86] and VSURF [87], on both fire modalities (“Fire” and “No-Fire” populations) and for all the cofactor-corrected flammability variables as well as for both fuel types. The choice was made to keep only the molecules that were common to both RF analyses (i.e., intersection).

Finally, the partial least squares (PLS) regression analyses were performed to determine the effect of the significant terpene molecules selected by the previous random forest analyses on each flammability variable. The significance of components in the resulting models was determined by uncertainty tests carried out within a full cross-validation. The scaled regression coefficients of the PLS models provided information on the effect (positive or negative) of each significant parameter (terpene compounds) on the flammability metrics, and its relative weight in the fitted model (absolute value) indicated the relative importance in predicting each flammability variable.

## 5. Conclusions

The cofactor-corrected flammability variables showed differences between the flammability of *P. halepensis* and *P. sylvestris* according to the fire modality, which were consistent with the fire adaptive strategies of these species. For the same fuel type, some less contrasted results obtained regarding species’ flammability underlined that it is important to consider not only a large range of flammability variables but also a large range of terpene compounds (i.e., analyses at the level of the terpene molecules and not just of the family). This was important to better assess global fuel flammability and the effects of these chemical compounds. The terpene contents were higher in the *P. halepensis’* “Fire” populations, suggesting that this trait could vary from one generation to the next in response to fire frequency as a selective pressure. The random forest analyses selected several groups of significant molecules that differed between the fire modalities for a given flammability variable for both species and fuel type. The terpene compounds driving flammability as well as their effects (mostly enhancing *P. halepensis*’ flammability in the “Fire” modality) also differed between the fire modalities (and also among the variables).

The importance of terpenes in stress tolerance is well known [47]. With ongoing climate change, terpene production in response to abiotic stresses (e.g., drought) and fire risk may simultaneously increase in a mutually reinforcing way. However, as there are other chemical compounds affecting plant flammability [42], it could be interesting to also study some of these compounds such as lignin or waxes that also are involved in the serotiny process [88,89]. In addition, knowing that serotinous cones are sealed by resins, it could be interesting to analyze if serotinous *P. halepensis* are more flammable compared to non-serotinous trees, and if the genes involved in the serotiny variation are involved in the terpene production. In order to better anticipate the impact of climate change and the resulting changes in fire regimes, improving our knowledge on the flammability variations, based on fire frequency as well as on the impact of chemical factors involved in this fuel flammability, represents a great challenge.

## Figures and Tables

**Table 1:** Summary results of the comparisons of *Pinus halepensis* shoot flammability between the two fire modalities (ANOVA) and the terpene compound associations (results of random forest analyses) corresponding to each variable of each modality. In italic font, the compounds highlighted as significantly affecting the variable and compounds in bold font: highest regression coefficient (results of PLS analyses). Variables in bold font: significant results between fire modalities. PHF: *Pinus halepensis* ”Fire” modality. PHNF: *Pinus halepensis* ”No-Fire” modality.

Flammability Variables	PHF	PHNF
**Time-to-ignition PNH > PH**	***α-terpineol***; cembrene	β-caryophyllene; cembrene; elemol; terpinolene; thujene
Maximum temperature	*α-humulene*; ***β-caryophyllene***; cembrene; germacrene-D; *limonene*; *thujene*	α-muurolene; ***α-terpineol***; β-cadinene; caryophyllene-oxide; cembrene; elemol
**Flaming duration PHF > PHNF**	α-pinene; α-terpinene; β-caryophyllene	α-muurolene; cembrene; ***valencene***
**Flame height PHNF > PHF**	α-muurolene; α-pinene; β-cadinene; *camphene*; caryophyllene-oxide; cembrene; ***elemol***; *γ-cadinene*; germacrene-D; limonene; ocimene	α-muurolene; ***camphene***; caryophyllene-oxide; *cembrene*; *valencene*
Radiative peak	*α-pinene*; α-humulene; *β-caryophyllene*; ***β-pinene***; *camphene*; caryophyllene-oxide; elemol; farnesen; *terpinolene*	α-muurolene; ***β-caryophyllene***; ***limonene***
Consumed weight	α-humulene; α-muurolene; α-terpinene; β-pinene; β-cadinene β-caryophyllene; caryophyllene-oxide; elemol; farnesen	*α-muurolene*; ***limonene***; *thunbergol*; valencene

**Table 2:** Summary results of the comparisons of *Pinus sylvestris*’ shoot flammability between the two fire modalities (ANOVA) and the terpene compound associations (results of random forest analyses) corresponding to each variable of each modality. In italic font, the compounds highlighted as significantly affecting the variable and compounds in bold font: highest regression coefficient (results of PLS analyses). Variables in bold font: significant results between fire modalities. PSF: *Pinus sylvestris* “Fire” modality. PSNF: *Pinus sylvestris* “No-Fire” modality.

SHOOTS	PSF	PSNF
Time-to-ignition	α-pinene; α-terpineol, tricyclene	δ-3-carene; humulene; unknown-sequi-35,46
**Maximum temperature PSF > PSNF**	α-muurolene; α-terpinene; β-myrcene; ***borneol***; *elemene*; *γ-terpinene*; unknown-sequi-34,5; tricyclene; valencene	δ-3-carene; ***α-terpineol***; *β-myrcene*; borneol; unknown-sequi-34,5; *unknown-sequi-35-46*
Flaming duration	borneol; camphor; elemene	-
Flame height	α-phellandrene; *α-terpineol*; *β-myrcene*; camphor; eucalyptol; ***tricyclene***; valencene	***Aromadendrene***; *unknown-sequi-35,46*; ylangene
**Radiative peak PSF > PSNF**	α-terpineol	-
Consumed weight	*δ-3-carene*; camphene; ***unknown-sequi-35,46***	-

**Table 3:** Summary results of the comparisons of *Pinus halepensis* litter flammability between the two fire modalities (ANOVA) and the terpene compound associations (results of random forest analyses) corresponding to each variable of each modality. In italic font, the compounds highlighted as significantly affecting the variable and compounds in bold font: highest regression coefficient (results of PLS analyses). Variables in bold font: significant results between fire modalities. PHF: *Pinus halepensis*’ “Fire” modality. PHNF: *Pinus halepensis*’ “No-Fire” modality.

	PHF	PHNF
**Time-to-ignition PHNF > PHF**	α-muurolene; *α-terpinene*; α-terpineol; farnesen; ***γ-terpinene***; *ocimene*; *thujene*	β-cadinene; cembrene; ***limonene***
Maximum temperature	*β-cadinene*; *camphene*; *elemol*; *ocimene*; *valencene*	***α-terpineol***; β-cadinene; *elemol*; germacrene-D; ocimene
**Flaming duration PHNF > PHF**	cembrene; *elemol*; γ-cadinene; germacrene-D; ***limonene***	α-muurolene; ***β-cadinene***; *cembrene*
**Flame height PHF > PHNF**	*α-pinene*; α-terpineol; *β-caryophyllene*; *camphene*; *germacrene-D*; ***ocimene***; valencene	***α-humulene***; *α-pinene*; α-terpineol; *β-caryophyllene*; camphene-; caryophyllene-oxide; limonene
**Rate of spread PHNF > PHF**	cembrene	camphene; caryophyllene-oxide; germacrene-D; limonene; ***terpinolene***; thujene; *valencene*
**Consumed weight PHF > PHNF**	***α-terpineol***; camphene; ocimene; valencene	*α-terpineol*; β-cadinene; *camphene*; ***caryophyllene-oxide***; cembrene; germacrene-D; terpinolene; valencene

**Table 4:** Summary results of the comparisons of *Pinus sylvestris* litter flammability between the two fire modalities (ANOVA) and the terpene compound associations (results of random forest analyses) corresponding to each variable of each modality. In italic font, the compounds highlighted as significantly affecting the variable and compounds in bold font: highest regression coefficient (results of PLS analyses). Variable in bold font: significant results between fire modalities. PSF: *Pinus sylvestris*’ “Fire” modality. PSNF: *Pinus sylvestris*’ “No-Fire” modality.

	PSF	PSNF
**Time-to-ignition PSF > PSNF**	***α-copaene***; α-terpineol; *β-bourbonene*; humulene; *linalool*	*α-terpineol*; ***linalool***; mix-sesquiterpene
**Maximum temperature PSF > PSNF**	δ-3-carene; α-bisabolol; *α-cadinene*; *α-cadinol*; ***α-copaene***; α-cubebene; α-terpinene; β-bourbonene; *borneol*; *camphor*; *elemene*; eucalyptol; humulene; linalool; unknown-sequi-35,17; *unknown-sequi-35,46*; *τ-cadinol*; ylangene	***α-phellandrene***; α-terpineol; *β-caryophyllene*; unknown-sequi-35,46
**Flame height PSNF > PSF**	*α-cadinol*; *α-terpineol*; β-bourbonene; elemene; eucalyptol; humulene; *linalool*; ocymene; unknown-sequi-35,17; ***τ-cadinol***; *tricyclene*	δ-3-carene; β-myrcene; β-caryophyllene; caryophyllene-oxide; linalool; nerol; tricyclene
**Flaming duration PSNF > PSF**	*α-terpineol*; β-bourbonene; ***borneol***; *linalool*; ylangene	-

## Data Availability

The datasets generated during and/or analyzed during the current study are available in the Zenodo repository, http://zenodo.org/deposit/5560927 (accessed on 8 October 2021).

## Supplementary Materials

The following supplementary data are available online at https://www.mdpi.com/article/10.3390/plants10102164/s1, Table S1. Relationships between flammability variables and flammability cofactors for shoots of *P. halepensis* and *P. sylvestris*; Table S2. Cofactors significantly related to each shoot flammability variable and results of one-way ANOVA performed on each corrected- flammability variable testing the difference between the two fire modalities for both species; Table S3. Relationships between flammability variables and flammability cofactors for litter of *P. halepensis* and *P. sylvestris*; Table S4. Cofactor significantly related to each litter flammability variable and results of one-way ANOVA performed on corrected- flammability variable (i.e., residuals extracted) testing the difference between both fire modalities for both species; Table S5. Terpene molecules identified in *P. halepensis* with their chemical formula and their concentration according to the fire modality; Table S6. Terpene molecules identified in *P. sylvestris* with their chemical formula and their concentration according to the fire modality; Table S7. Terpene molecules related to each flammability variable according to the fire modality for *P. halepensis*’ shoot; TableS8. Terpene molecules related to each flammability variable according to the fire modality for *P. halepensis’* litter; Table S9. Terpene molecules related to each flammability variable according to the fire for *P. sylvestris’* shoot; Table S10. Terpene molecules related to each flammability variable according to the fire modality for *P. sylvestris’* litter; Table S11. Partial least squares (PLS) regression analyses performed on both fire modalities and each *P. halepensis*’ shoots flammability variables highlighting the scaled regression coefficients (R) of the significant terpene molecules; Table S12. Partial least squares (PLS) regression analyses performed on both fire modalities and each *P. halepensis’* litter flammability variables and highlighting the scaled regression coefficients (R) of the significant terpene molecules; Table S13. Partial least squares (PLS) regression analyses performed on both fire modalities and each *P. sylvestris’* shoot flammability variables and highlighting the scaled regression coefficients (R) of the significant terpene molecules; Table S14. Partial least squares (PLS) regression analyses performed on both fire modalities and each *P. sylvestris’* litter flammability variables and highlighting the scaled regression coefficients (R) of the significant terpene molecules.
