# Supplementary material for: Effect of Fire Frequency on the Flammability of Two Mediterranean Pines: Link with Needle Terpene Content
Source: Plants (Basel). 2021 Oct 12;10(10):2164. doi: 10.3390/plants10102164 (PMC8541587; doi:10.3390/plants10102164)
Supplement: Supplementary file 1 [file plants-10-02164-s001.zip › plants-1365952-supplementary.pdf]

SUPPLEMENTARY MATERIALS

Table S1**.** Relationships between flammability variables (TTi: time-to-ignition; CW: Consumed weight; Tmax: Maximum temperature; FD: Flaming duration; FH: Flame height; RP: radiative peak) and flammability cofactors (SVR: Surface-to-volume ratio; FMC: Fuel moisture content) for shoots of *P. halepensis* and *P. sylvestris*. (One-way ANOVA; F = Fisher test, p = probability, NS= non significant).

Shoots	*P.halepensis*			*P. sylvestris*		
	SVR	Bulk density	FMC	SVR	Bulk density	FMC
TTi	F= 20.7 p< 0.0001	NS	F= 43.4 p< 0.0001	F= 23.5 p< 0.0001	NS	F= 21.5 p< 0.0001
CW	F= 4.5 p= 0.035	F= 4.4 p= 0.027	F= 4.2 p= 0.042	F= 10.0 p= 0.002	NS	F= 8.0 p= 0.0052
Tmax	F= 4.5 p= 0.035	F= 4.9 p= 0.027	F= 67.4 p< 0.0001	F= 7.8 p= 0.006	F= 133.7 p< 0.0001	F= 27.7 p< 0.0001
FD	F= 5.7 p= 0.017	F= 4.4 p= 0.036	NS	F= 25.5 p< 0.0001	NS	NS
FH	NS		F= 130.2 p< 0.0001	F= 18.3 p< 0.0001	F= 11.4 p= 0.0009	F= 10.2 p= 0.002
RP	NS	F= 14.8 p= 0.0001	F= 5.7 p= 0.018	NS	F= 9.9 p= 0.002	F= 5.5 p= 0.020

Table S2. Cofactors (SVR: surface-to-volume ratio; FMC: fuel moisture content; BD: bulk density) significantly related to each shoot flammability variable (using simple linear regression analyses) and results of one-way ANOVA (F = Fisher test, p = probability) performed on each corrected- flammability variable testing the difference between the two fire modalities for both species. In bold: significant results. PHF= *P. halepensis* "Fire" modality, PHNF= *P. halepensis* "No-Fire" modality, PSF= *P. sylvestris* "Fire" modality and PSNF= *P. sylvestris* "No-Fire" modality. N= 380 for *P. halepensis* and N= 293 for *P. sylvestris*.

Shoots	*P. halepensis*		*P. sylvestris*	
	Significant cofactors	ANOVA results on corrected flammability variable	Significant cofactors	ANOVA results on corrected flammability variable
Time-to-ignition	SVR; FMC	**F= 17.42 ; p < 0.0001 PHF < PHNF**	FMC; SVR	F= 0.75 ; p= 0.3866
Consumed weight	SVR; BD; FMC	F= 2.59 ; p = 0.1083	FMC; SVR	F= 2.13 ; p= 0.1453
Maximum temperature	FMC; SVR; BD	F= 0.00 ; p= 0.9607	FMC; BD; SVR	**F= 25.28 ; p < 0.0001 PSF > PSNF**
Flaming duration	SVR; BD	**F= 8.90 ; p= 0.0030 PHF > PHNF**	SVR	F= 0.65 ; p= 0.4200
Flame height	FMC	**F= 10.89 ; p= 0.0011 PHF < PHNF**	SVR; BD; FMC	F= 2.35 ; p= 0.1263
Radiative peak	BD; FMC	F= 2.35 ; p= 0.1263	FMC; BD	**F= 11.92 ; p= 0.0006 PSF > PSNF**

Table S3. Relationships between flammability variables (TTi: time to ignition; CW: Consumed weight; Tmax: Maximum temperature; FD Flaming duration; FH: Flame height) and flammability cofactors (SVR: Surface-to-volume ratio) for litter of *P. halepensis* and *P. sylvestris*.

**Litter**	***P. halepensis***	***P. sylvestris***
	SVR	SVR
TTi	NS	F= 20.1 p< 0.0001
CW	NS	-
Tmax	NS	NS
FD	F= 6.1 p= 0.014	F= 4.6 p= 0.033
FH	F= 5.7 p= 0.018	F= 4.5 p= 0.035

Table S4**.** Cofactor (SVR: surface-to-volume ratio) significantly related to each litter flammability variable (using simple linear regression analyses; NS when non-significant) and results of one-way ANOVA (F = Fisher test, p = probability and KW = Kruskal-Wallis test, p = probability) performed on corrected-flammability variable (i.e. residuals extracted) testing the difference between both fire modalities for both species. In bold: significant results. PHF= *P. halepensis* "Fire" modality, PHNF= *P. halepensis* "No-Fire" modality, PSF= *P. sylvestris* "Fire" modality and PSNF= *P. sylvestris* "No-Fire" modality. N= 344 for *P. halepensis* and N= 279 for *P. sylvestris*. For *P. sylvestris*, consumed weight was 100% and the rate of spread was not measured so these variables were not taken into account in the analyses.

Litter	*P.halepensis*		*P. sylvestris*	
	Significant cofactors	ANOVA results on corrected flammability variable	Significant cofactors	ANOVA results on corrected flammability variable
Time-to-ignition	NS	**F= 34.48 ; p < 0.0001<br>PHF < PHNF**	SVR	**KW = 10.63 ; p= 0.001<br>PSF > PSNF**
Consumed weight	NS	**F= 10.17 ; p= 0.0016<br>PHF > PHNF**	-	-
Maximum temperature	NS	F= 0.01 ; p= 0.9222	NS	**F= 23.09 ; p< 0.0001<br>PSF > PSNF**
Flaming duration	SVR	**F= 5.23 ; p= 0.0232<br>PHF < PHNF**	SVR	**KW = 18.94 ; p< 0.0001<br>PSF < PSNF**
Flame height	SVR	**KW = 21.93 ; p < 0.0001<br>PHF > PHNF**	SVR	**F= 107.38 ; p< 0.0001<br>PSF < PSNF**
Rate of spread	NS	**F= 14.75 ; p= 0.0001<br>PHF < PHNF**	-	-

Table S5. Terpene molecules identified in *P. halepensis* with their chemical formula and their concentration (mg g^{-1} DM; mean ± standard deviation) according to the fire modality.

	Formule	"Fire"	"No-Fire"
Thujene	C10H16	0.050 (±0.044)	0.038 (±0.040)
α-pinene	C10H16	0.737 (±0.415)	0.663 (±0.463)
Camphene	C10H16	0.008 (±0.004)	0.007 (±0.005)
β-pinene	C10H16	0.428 (±0.321)	0.363 (±0.308)
δ-3-carene	C10H16	0.114 (±0.112)	0.092 (±0.094)
α-terpinene	C10H16	0.010 (±0.009)	0.008 (±0.009)
Limonene	C10H16	0.087 (±0.084)	0.077 (±0.078)
Ocimene	C10H16	0.069 (±0.056)	0.059 (±0.057)
ϒ-terpinene	C10H16	0.042 (±0.033)	0.035 (±0.033)
Terpinolene	C10H16	0.506 (±0.348)	0.442 (±0.384)
α-terpineol	C10H18O	0.014 (±0.010)	0.016 (±0.016)
β-caryophyllene	C15H24	2.555 (±1.130)	2.201 (±1.228)
α-humulene	C15H24	0.406 (±0.208)	0.342 (±0.206)
Farnesene	C15H24	0.223 (±0.121)	0.186 (±0.105)
Germacrene-D	C15H24	0.037 (±0.032)	0.033 (±0.032)
Valencene	C15H24	0.118 (±0.140)	0.910 (±0.132)
α-muurolene	C15H24	0.609 (±0.969)	0.602 (±1.220)
ϒ-cadinene	C15H24	0.068 (±0.063)	0.049 (±0.053)
β-cadinene	C15H24	0.317 (±0.407)	0.265 (±0.385)
Elemol	C15H26O	1.053 (±1.231)	0.837 (±1.103)
Caryophyllene-oxide	C15H24O	0.772 (±0.815)	0.590 (±0.598)
Cembrene	C20H32	0.441 (±0.703)	0.293 (±0.530)
Thunbergol	C20H34O	0.424 (±0.641)	0.324 (±0.525)

Table S6. Terpene molecules identified in *P. sylvestris* with their chemical formula and their concentration (mg g^{-1} DM; mean ± standard deviation) according to the fire modality.

	Formule	"Fire"	"No-Fire"
Tricyclene	C10H16	0.394 (±0.40)	0.406 (±0.404)
α-pinene	C10H16	2.790 (±1.072)	1.072 (±1.266)
Camphene	C10H16	0.561 (±0.360)	0.535 (±0.307)
β-pinene	C10H16	0.382 (±0.310)	0.324 (±0.234)
β -Myrcene	C10H16	0.412 (±0.408)	0.438 (±0.485)
α-phellandrene	C10H16	0.002 (±0.006)	0.001 (±0.001)
δ-3-Carene	C10H16	0.056 (±0.183)	0.102 (±0.273)
α-terpinene	C10H16	0.001 (±0.002)	0.001 (±0.002)
Ocimene	C10H14	0.001 (±0.001)	< 0.001
Limonene	C10H16	0.053 (±0.037)	0.050 (±0.035)
Eucalyptol	C10H18O	0.004 (±0.007)	0.003 (±0.004)
β-ocimene	C10H16	0.156 (±0.099)	0.165 (±0.081)
ϒ-terpinene	C10H16	0.006 (±0.008)	0.007 (±0.009)
Terpinolene	C10H16	0.019 (±0.037)	0.016 (±0.024)
Linalool	C10H18O	0.001 (±0.002)	0.001 (±0.002)
Camphor	C10H16O	0.003 (±0.004)	0.004 (±0.004)
Borneol	C10H18O	0.010 (±0.012)	0.010 (±0.012)
α-terpineol	C10H18O	0.009 (±0.014)	0.007 (±0.007)
Nerol	C10H18O	< 0.001	< 0.001
Elemene	C15H24	0.092 (±0.135)	0.075 (±0.098)
α-cubebene	C15H24	0.168 (±0.216)	0.185 (±0.217)
Ylangene	C15H24	0.023 (±0.036)	0.033 (±0.069)
α-copaene	C15H24	0.195 (±0.245)	0.207 (±0.247)
β-bourbonene	C15H24	0.015 (±0.022)	0.017 (±0.023)
β -cubebene	C15H24	0.050 (±0.038)	0.048 (±0.036)
β -elemene	C15H24	0.033 (±0.033)	0.025 (±0.028)
Longifolene	C15H24	< 0.001	0.001 (±0.001)
β -caryophyllene	C15H24	0.405 (±0.176)	0.443 (±0.220)
Aromandendrene	C15H24	0.024 (±0.027)	0.024 (±0.028)
Humulene	C15H24	0.066 (±0.043)	0.072 (±0.047)
sesqui unk_34,5	C15-	0.101 (±0.102)	0.084 (±0.095)
sesqui unk_35,17	C15-	0.185 (±0.428)	0.187 (±0.345)

sesqui unk_35,28	C15-	0.375 (±0.476)	0.339 (±0.380)
sesqui unk_35,46	C15-	0.020 (±0.023)	0.021 (±0.024)
Valencene	$C_{15}H_{24}$	0.047 (±0.047)	0.044 (±0.048)
α -Muurolene	$C_{15}H_{24}$	0.066 (±0.066)	0.071 (±0.070)
ϒ -cadinene	$C_{15}H_{24}$	0.463 (±0.310)	0.451 (±0.323)
β -cadinene	$C_{15}H_{24}$	0.519 (±0.329)	0.483 (±0.311)
α -cadinene	$C_{15}H_{24}$	0.037 (±0.045)	0.037 (±0.044)
α -bisabolol	$C_{15}H_{26}O$	0.006 (±0.011)	0.008 (±0.013)
mix cyclic sesquiterpene alcohol	C15-	0.243 (±0.187)	0.243 (±0.246)
Caryophyllene oxide	$C_{15}H_{24}O$	0.381 (±0.395)	0.454 (±0.546)
τ -cadinol	$C_{15}H_{26}O$	0.089 (±0.119)	0.092 (±0.130)
α -cadinol	$C_{15}H_{26}O$	0.038 (±0.049)	0.036 (±0.049)

Table S7. Terpene molecules related to each flammability variable according to the fire modality (PH: *Pinus halepensis;* F: fire modality; NF: no-fire modality) for *P. halepensis*' shoot. Terpene molecules were considered as related to flammability when they were at the intersect of the two types of random forest analyses (i.e. VSURF and Boruta). In bold: common molecules to both fire modalities.

	**PHF**	**PHNF**
Time-to-ignition	α-terpineol; **cembrene**	β-caryophyllene; **cembrene**; elemol; terpinolene ; thujene
Maximum temperature	α-humulene; β-caryophyllene; **cembrene** ; germacrene-D ; limonene ; thujene	α-muurolene; α-terpineol; β-cadinene; caryophyllene-oxide; **cembrene**; elemol
Flaming duration	α-pinene; α-terpinene; β-caryophyllene	α-muurolene; cembrene; valencene
Flame height	**α-muurolene**; α-pinene; β-cadinene; **camphene**; **caryophyllene-oxide**; **cembrene**; elemol; γ-cadinene; germacrene-D; limonene; ocimene	α-muurolene; camphene; caryophyllene-oxide; cembrene; valencene
Radiative peak	α-pinene; α-humulene; **β-caryophyllene**; β-pinene; camphene; caryophyllene-oxide; elemol; farnesene; terpinolene	α-muurolene; **β-caryophyllene**; limonene
Consumed weight	α-humulene; **α-muurolene**; α-terpinene; β-pinene; β-cadinene β-caryophyllene; caryophyllene-oxide; elemol; farnesene	**α-muurolene**; limonene; thunbergol; valencene

Table S**8.** Terpene molecules related to each flammability variable according to the fire modality (PH: *Pinus halepensis;* F: fire modality; NF: no-fire modality) for *P. halepensis'* litter. Terpene molecules were considered as related to flammability when they were at the intersect of the two types of random forest analyses (i.e. VSURF and Boruta). In bold: common molecules to both fire modalities.

	**PHF**	**PHNF**
Time-to-ignition	α-muurolene; α-terpinene; α-terpineol ; farnesene ; γ-terpinene; ocimene; thujene	β-cadinene; cembrene; limonene
Maximum temperature	**β-cadinene** ; camphene ; **elemol** ; ocimene; valencene	α-terpineol; **β-cadinene**; **elemol**; germacrene-D; ocimene
Flaming duration	**cembrene**; elemol; γ-cadinene; germacrene-D; limonene	α-muurolene; β-cadinene; **cembrene**
Flame height	**α-pinene; α-terpineol**; **β-caryophyllene**; **camphene**; germacrene-D; ocimene; valencene	α-humulene; **α-pinene**; **α-terpineol** ; **β-caryophyllene; camphene**; caryophyllene-oxide; limonene
Rate of spread	cembrene	camphene; caryophyllene-oxide; germacrene-D; limonene; terpinolene; thujene; valencene
Consumed weight	**α-terpineol**; **camphene**; ocimene; **valencene**	**α-terpineol**; β-cadinene; **camphene**; caryophyllene-oxide; cembrene; germacrene-D; terpinolene; **valencene**

Table S**9.** Terpene molecules related to each flammability variable according to the fire modality (PS: *Pinus sylvestris;* F: fire modality; NF: no-fire modality) for *P. sylvestris'* shoot. Terpene molecules were considered as related to flammability when they were at theintersect of the two types of random forest analyses (i.e. VSURF and Boruta). In bold: common molecules to both fire modalities.

	**PSF**	**PSNF**
Time-to-ignition	α-pinene; α-terpineol, tricyclene	Δ-3-carene; humulene; unknown-sequi-35,46
Maximum temperature	α-muurolene; α-terpinene; β-myrcene; **borneol**; elemene; γ-terpinene; **unknown-sequi-34,5;** tricyclene; valencene	Δ-3-carene; α-terpineol; β-myrcene; **borneol; unknown-sequi-34,5**; unknown-sequi-35,46
Flaming duration	borneol; camphor; elemene	-
Flame height	α-phellandrene; α-terpineol; β-myrcene; camphor; eucalyptol; tricyclene; valencene	aromadendrene; unknown-sequi-35,46; ylangene
Radiative peak	α-terpineol	-
Consumed weight	δ-3-carene; camphene; unknown-sequi-35,46	-

Table S**10.** Terpene molecules related to each flammability variable according to the fire modality (PS: *Pinus sylvestris;* F: fire modality; NF: no-fire modality) for *P. sylvestris'* litter. Terpene molecules were considered as related to flammability when they were at the intersect of the two types of random forest analyses (i.e. VSURF and Boruta). In bold: common molecules to both fire modalities.

	**PSF**	**PSNF**
Time-to-ignition	α-copaene; **α-terpineol**; β-bourbonene; humulene; linalool	**α-terpineol**; linalool; mix-sesquiterpene
Maximum temperature	δ-3-carene; α-bisabolol; α-cadinene; α-cadinol; α-copaene; α-cubebene; α-terpinene; β-bourbonene; borneol; camphor; elemene; eucalyptol; humulene; linalool; unknown-sequi-35,17; **unknown-sequi-35,46**; τ-cadinol; ylangene	α-phellandrene; α-terpineol; β-caryophyllene; **unknown-sequi-35,46**
Flame height	α-cadinol; α-terpineol; β-bourbonene; elemene; eucalyptol; humulene; **linalool**; ocimene; unknown-sequi-35,17; τ-cadinol; **tricyclene**	δ-3-carene; β-myrcene; β-caryophyllene; caryophyllene-oxide; **linalool**; nerol**; tricyclene**
Flaming duration	α-terpineol; ; β-bourbonene; borneol; linalool; ylangene	-

Table S**11.** Partial least square (PLS) regression analyses (p= p value, R^2= determination coefficient, Cp= number of component) performed on both fire modalities (PH: *Pinus halepensis*; F: fire modality; NF: no-fire modality) and each *P. halepensis*' shoots flammability variables highlighting the scaled regression coefficients (R) of the significant terpene molecules. In bold: main flammability driver. MT: Monoterpenes; ST: Sesquiterpenes; DT: Diterpenes.

	**PHF**		**PHNF**	
Time-to-ignition	**α-terpineol (MT)**	**R= 0.43**	NS	
	p< 0.0001 ; R^2= 0.18 ; Cp = 1			
Maximum temperature	α-humulene (ST)	R= -1,65	**α-terpineol (MT)**	**R= 0.66**
	**β-caryophyllene (ST)**	**R= 2.17**	p< 0.0001 ; R^2= 0.44 ; Cp = 1	
	limonene (MT)	R= -0.31		
	thujene (MT)	R= 0.28		
	p< 0.0001 ; R^2= 0.50 ; Cp = 4			
Flaming duration	NS		**valencene (ST)**	**R= -0.24**
			p= 0.002 ; R^2= 0.06 ; Cp = 1	
Flame height	camphene (MT)	R= -0.47	**camphene (MT)**	**R= -0.53**
	**elemol (ST)**	**R= 2.24**	cembrene (DT)	R= 0.23
	γ-cadinene (ST)	R= 0.30	valencene (ST)	R= 0.28
	p< 0.0001 ; R^2= 0.51 ; Cp = 2		p< 0.0001 ; R^2= 0.45 ; Cp = 2	
Radiative peak	camphene (MT)	R= 0.22	**β-caryophyllene (ST)**	**R= 0.29**
	α-pinene (MT)	R= -0.52	**limonene (MT)**	**R= -0.29**
	β-caryophyllene (ST)	R= 0.51	p= 0.003 ; R^2= 0.07 ; Cp = 2	
	**β-pinene (MT)**	**R= 0.61**		
	terpinolene (MT)	R= -0.52		
	p< 0.0001 ; R^2= 0.24 ; Cp = 5			
Consumed weight	NS		α-humulene (ST)	R= 0.30
			**limonene (MT**	**R= -0.31**
			thunbergol (DT)	R= -0.24
			p= 0.002 ; R^2= 0.09 ; Cp = 3	

Table S**12.** Partial least square (PLS) regression analyses (p= p value, R^2= determination coefficient, Cp= number of component, NS=non-significant) performed on both fire modalities (PH: *Pinus halepensis*; F: fire modality; NF: no-fire modality) and each *P. halepensis'* litter flammability variables and highlighting the scaled regression coefficients (R) of the significant terpene molecules. In bold: main flammability driver. MT: Monoterpenes; ST: Sesquiterpenes; DT: Diterpenes.

	**PHF**	**PHNF**
Time-to-ignition	α-terpinene (MT) R= 0,55<br>**γ-terpinene (MT) R= -1.05**<br>ocimene (MT) R= -0.34<br>thujene (MT) R= 0.60<br>p< 0.0001 ; R^2= 0.14 ; Cp = 4	**limonene (MT) R= -0.25**<br>p= 0.003 ; R^2= 0.06 ; Cp = 1
Maximum temperature	**β-cadinene (ST) R= -0.62**<br>camphene (MT) R= 0.42<br>elemol (ST) R= 0.52<br>ocimene (MT) R= -0.39<br>valencene (ST) R= -0.30<br>p< 0.0001 ; R^2= 0.57 ; Cp = 4	**α-terpineol (MT) R= 0.57**<br>elemol (ST) R= -0.20<br>p< 0.0001 ; R^2= 0.44 ; Cp = 1
Flaming duration	elemol (ST) R= 0.20<br>**limonene (MT) R= 0.30**<br>p< 0.0001 ; R^2= 0.21 ; Cp = 1	**β-cadinene (ST) R= -0.24**<br>cembrene (DT) R= -0.22<br>p< 0.0001 ; R^2= 0.17 ; Cp = 1
Flame height	α-pinene (MT) R= 0.27<br>β-caryophyllene (ST) R= 0.33<br>camphene (MT) R= -0.33<br>germacrene D (ST) R= -0.34<br>**ocimene (MT) R= -0.40**<br>p< 0.0001 ; R^2= 0.21 ; Cp = 5	**α-humulene (ST) R= -0.49**<br>α-pinene (MT) R= -0.23<br>β-caryophyllene (ST) R= 1.18<br>p< 0.0001 ; R^2= 0.33 ; Cp = 2
Rate of spread	NS	**terpinolene (MT) R= 0.27**<br>valencene (ST) R= -0.26<br>p= 0.003 ; R^2= 0.11 ; Cp = 2
Consumed weight	**α-terpineol (MT) R= -0.47**<br>p< 0.0001 ; R^2= 0.22 ; Cp = 1	α-terpineol (MT) R= -0.23<br>camphene (MT) R= -0.22<br>**caryophyllene-oxide (ST) R= 0.28**<br>p< 0.0001 ; R^2= 0.27 ; Cp = 1

Table S**13.** Partial least square (PLS) regression analyses (p= p value, R^2= determination coefficient, Cp= number of component, NS=non-significant) performed on both fire modalities (PS: *Pinus sylvestris*; F: fire modality; NF: no-fire modality) and each *P. sylvestris'* shoot flammability variables and highlighting the scaled regression coefficients (R) of the significant terpene molecules. In bold: main flammability driver. MT: Monoterpenes; ST: Sesquiterpenes.

	PSF	PSNF
Time-to-ignition	NS	NS
Maximum temperature	**borneol (MT) R= 0.41**<br>elemene (ST) R= -0.25<br>γ-terpinene (MT) R= -0.26<br>p< 0.0001 ; R^2= 0.60 ; Cp = 3	**α-terpineol (MT) R= 0.35**<br>β-myrcene (MT) R= -0.25<br>unknown-sequi-35,46 (ST) R= -0.31<br>p< 0.0001 ; R^2= 0.62 ; Cp = 1
Flaming duration	NS	NS
Flame height	α-terpineol (MT) R= -0.22<br>β-myrcene (MT) R= 0.22<br>**tricyclene (MT) R= 0.24**<br>p< 0.0001 ; R^2= 0.24 ; Cp = 2	**aromadendrene (ST) R= 0.23**<br>unknown-sequi-35,46 (ST) R= 0.22<br>p< 0.0001 ; R^2= 0.18 ; Cp = 1
Radiative peak	NS	NS
Consumed weight	δ-3-carene (MT) R= 0.20<br>**unknown-sequi-35,46 (ST) R= 0.30**<br>p< 0.0001 ; R^2= 0.14 ; Cp = 1	NS

Table S**14.** Partial least square (PLS) regression analyses (p= p value, R^2= determination coefficient, Cp= number of component, NS=non-significant) performed on both fire modalities modalities (PS: *Pinus sylvestris*; F: fire modality; NF: no-fire modality) and each *P. sylvestris'* litter flammability variables and highlighting the scaled regression coefficients (R) of the significant terpene molecules. In bold: main flammability driver. MT: Monoterpenes; ST: Sesquiterpenes.

	PSF	PSNF
Time-to-ignition	**α-copaene (ST) R= 0.24**<br>β-bourbonene (ST) R= 0.21<br>linalool (MT) R= 0.22<br>$p < 0.0001$; R^2= 0.31 ; Cp = 1	α-terpineol (MT) R= -0.22<br>**linalool (MT) R= 0.23**<br>$p < 0.0001$; R^2= 0.16 ; Cp = 2
Maximum temperature	τ -cadinol (ST) R= 0.39<br>α-cadinene (ST) R= 0.37<br>α-cadinol (ST) R= -0.47<br>**α-copaene (ST) R= -0.51**<br>borneol (MT) R= 0.25<br>camphor (MT) R= 0.31<br>elemene (ST) R= -0.38<br>unknown-sequi-35,46 (ST) R= 0.29<br>$p < 0.0001$; R^2= 0.57 ; Cp = 8	**α-phellandrene (ST) R= -0.24**<br>β-caryophyllene (ST) R= 0.21<br>p= 0.0006 ; R^2= 0.11 ; Cp = 1
Flaming duration	α-terpineol (MT) R= 0.26<br>**borneol (MT) R= 0.37**<br>linalool (MT) R= -0.32<br>$p < 0.0001$; R^2= 0.41 ; Cp = 2	NS
Flame height	**τ-cadinol (ST) R= 0.47**<br>α-cadinol (ST) R= -0.25<br>α-terpineol (MT) R= -0.28<br>linalool (MT) R= 0.20<br>tricyclene (MT) R= -0.34<br>$p < 0.0001$; R^2= 0.45 ; Cp = 5	NS
